# Pure Testicular Seminoma Relapsing Late with Somatic Type Malignancy

**DOI:** 10.1155/2017/2457023

**Published:** 2017-03-07

**Authors:** Klaus-Peter Dieckmann, Petra Anheuser, Ralf Gehrckens, Waldemar Wilczak, Guido Sauter, Doris Höflmayer

**Affiliations:** ^1^Department of Urology, Albertinen-Krankenhaus Hamburg, Süntelstrasse 11a, 22457 Hamburg, Germany; ^2^Department of Diagnostic Radiology, Albertinen-Krankenhaus Hamburg, Süntelstrasse 11a, 22457 Hamburg, Germany; ^3^Institute of Pathology, Universitätsklinikum Hamburg-Eppendorf, Martinistrasse 52, 20246 Hamburg, Germany

## Abstract

*Background. *Somatic type malignancy (STM) occurs in 2% of all germ cell tumours (GCTs). The prognosis is unfavourable and the origin is poorly understood. Pathogenetic hypotheses involve direct transformation of teratoma, origin from totipotent cancer cells, or derivation from yolk sac tumour elements.* Case Presentation. *A 31-year-old patient was cured from testicular seminoma clinical stage IIc by orchiectomy and cisplatin-based chemotherapy. Nine years later, he experienced a late relapse with a mass sized 5 × 6 cm located at the former metastatic site. As no remission occurred after chemotherapy with three cycles of cisplatin, ifosfamide and etoposide, the mass was surgically resected. Histologically, the specimen consisted of neurofibroma with areas of malignant peripheral nerve sheath tumour and spots with mature bone formation. FISH analysis disclosed isochromosome 12p in the majority of evaluated cells suggesting somatic type malignancy (STM) of GCT. The patient is well 1 year after surgery.* Conclusion. *The pathogenesis of this STM remains enigmatic. The origin from GCT was evidenced by documentation of isochromosome 12p. Unrecognized teratomatous elements in the primary and totipotent cancer cells surviving the first chemotherapy could be hypothesized to represent the origin. STM developing from seminoma cells would be another novel hypothesis.

## 1. Background

Germ cell tumours (GCTs) have the potential to differentiate into many directions [1, 2]. The most surprising morphologic variant is the somatic type malignancy (STM) formerly called malignant transformation. Friedman and Moore were the first to document cases with “neuroepithelioma” and sarcoma arising within teratomatous germ cell tumours [3]. Pathologically, STMs encompass a wide spectrum of histologic appearances [4] with sarcoma of all subtypes comprising 50% and carcinomas of various subtypes comprising about 20% of cases followed by primitive neuroectodermal tumours (PNET) ranking third with 10% [5, 6]. The remainder is made up of rare malignancies including haematologic neoplasms and undifferentiated tumours [4, 7].

Morphologically, STMs appear like typical primary neoplasms with no obvious histological resemblance to germ cell tumours. However, modern FISH (fluorescence in situ hybridization) techniques have revealed that most of the STMs have retained isochromosome 12p which is characteristic for GCTs despite their morphologic dissimilarity [8].

STMs are rare events that are encountered in about 3% of metastasized cases with GCT [5, 9, 10]. Prognosis is usually unfavourable because cisplatin-based chemotherapy is not efficacious in these histologic variants [11]. Surgical excision represents the mainstay of treatment in these cases. After the first report on a small series of STM with documentation of clinical outcome in 1985 [12], no more than six systematic clinicopathological evaluations and several small case series have been reported to date encompassing around 500 cases with STM [5, 9, 10, 13–18]. Accordingly, the knowledge regarding incidence, biological behavior, and clinical management is still limited at present. We here report the case of a patient with STM that is exceptional because of its clinical and histologic features.

## 2. Case Description

**Figure 1:**
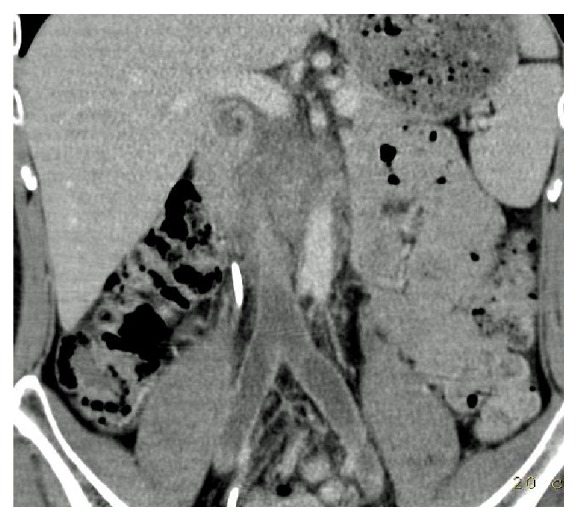
Abdominal computerized tomography showing a large mass in the upper retroperitoneum compressing the vena cava. Thrombotic occlusion of vena cava with extension of intravascular clotting into both iliac veins.

**Figure 2:**
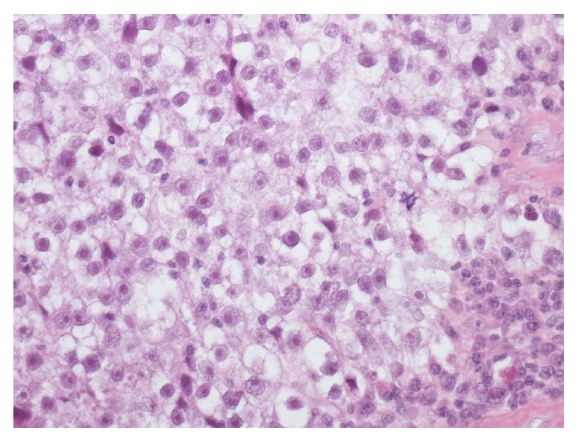
Histological section of the orchiectomy specimen showing pure seminoma. Haematoxylin-eosin stain, original ×100.

**Figure 3:**
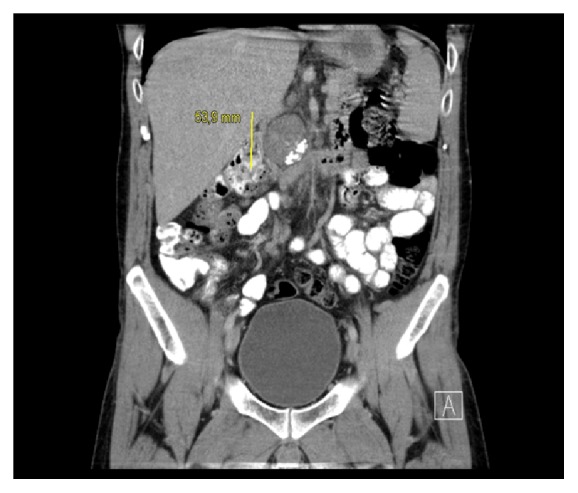
Abdominal CT scan showing a 5 × 6 cm mass with several calcifications between abdominal aorta and vena cava below the left renal vein: location is identical with metastatic site at first presentation.

A 31-year-old white man of European ancestry who was on insulin treatment for diabetes mellitus type 1 since childhood presented with right-sided back pain. Computed tomography (CT) disclosed a 7 × 11 cm retroperitoneal mass compressing the vena cava and additional mediastinal lymphadenopathy of 2.1 cm in size. Further clinical evaluation revealed a right-sided testicular mass and a thrombosis of the vena cava with extension of the intravascular clotting into both iliac veins (Figure 1). The serum level of beta human chorionic gonadotropin (bHCG) was increased to 36.6 U/L (upper limit of norm, 2.5 U/L); alpha fetoprotein (AFP) and lactate dehydrogenase (LDH) were within normal limits. The management consisted of antithrombotic treatment and inguinal orchiectomy. Histologic evaluation revealed pure testicular seminoma (Figure 2). Subsequently, the patient received chemotherapy with 3 courses of cisplatin, etoposide, and bleomycin. Upon restaging, the bHCG serum level had normalized and the mediastinal mass had disappeared, while the retroperitoneal mass had shrunk to 3 × 3 cm. One year thereafter, the residual mass had completely disappeared. With respect to the thrombotic occlusion of the vena cava, multiple collateral veins had evolved in the retroperitoneum. Later, during follow-up, the patient developed hypogonadism with the need for testosterone replacement therapy. Nine years after initial treatment, the patient presented with abdominal pain. Abdominal CT revealed an oval mass sized 5 × 6 cm located in the retroperitoneum caudal to the left renal vein and between vena cava and abdominal aorta (Figure 3). No other neoplastic deposits were observed. The serum levels of bHCG, AFP, and LDH were within normal limits. The findings were considered to represent a late relapse of seminoma mainly because the newly evolved mass was located right at the site of the initial metastasis 9 years priorly. Accordingly, the patient received a chemotherapy regimen consisting of three cycles of cisplatin, ifosfamide, and etoposide. Upon restaging with CT, no significant change of the retroperitoneal mass was noted. Therefore, surgical excision was performed. Despite the complex intraabdominal venous collateral network, a complete surgical excision of the mass was accomplished. Postoperative recovery was uneventful, and, 6 months thereafter, the patient is in good condition and is pursuing his work again.

**Figure 4:**
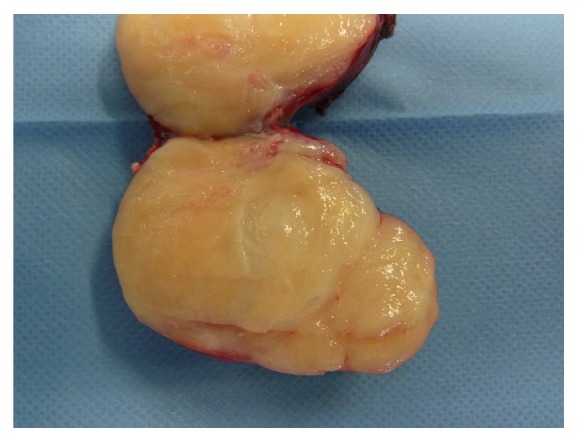
Cut section of the retroperitoneal mass after surgical excision: firm white-greyish mass and a clearly demarcated rim.

**Figure 5:**
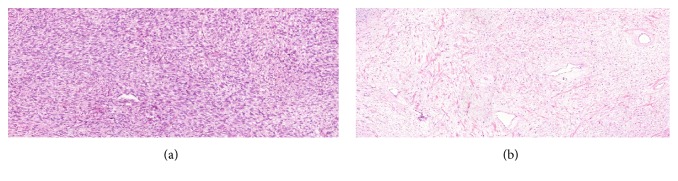
(a) Histological section of the resected abdominal mass: representative images of the tumour, composed of dispersed cells with no atypia, sometimes with a background of collagen fibers, resembling neurofibroma. Haematoxylin-eosin stain, original ×50. (b) Histological section of the resected abdominal mass: More hypercellular areas with moderate nuclear atypia, resembling a malignant peripheral nerve sheath tumour (MPNST). Haematoxylin-eosin stain, original ×50.

**Figure 6:**
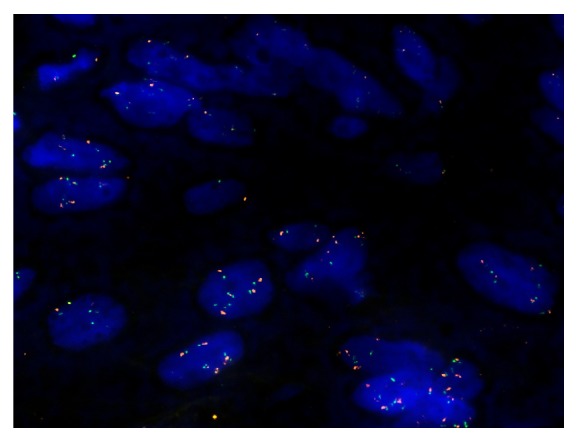
FISH analysis of resected abdominal mass: dual color probe (ZytoLight KRAS/CEN 12), demonstrating polysomy and isochromosome 12p (CEN 12: red; KRAS: green); magnification 1000x.

Cut section of the specimen revealed a firm white-greyish mass with several bone-like appearing tiny spots and a clearly demarcated rim (Figure 4). Histological evaluation disclosed loosely whorled and short intersecting fascicles composed of cells with round to oval nuclei, with small nucleoli and a small-to-moderate amount of mostly clear cytoplasm. Along the edge, some mature bone formation was observed. Noteworthy, in several areas of the specimen, the cells were more crowded, the nuclei showed atypia, and mitotic activity was observed. Immunohistochemical stains with glial fibrillary astrocytic protein (GFAP) demonstrated positive cytoplasmic staining; S100 showed focal nuclear and cytoplasmic positive staining. Germ cell tumour markers (Oct3/4, PLAP, SALL4, Glypican 3, and CD30), pancytokeratin (AE1/AE3), and neurofilament muscle specific markers (Desmin) as well as CD34, CD117, SOX10, MDM2, and EMA were all negative. The proliferation marker Ki-67 was positive in less than 5% of cells. Given the overall rather benign neurogenic differentiation with focal increased cellularity, nuclear atypia, and mitotic activity, the diagnosis of neurofibroma with transition into a low-grade malignant peripheral nerve sheath tumour (MPNST) was made (Figures 5(a) and 5(b)). No components of seminoma or other germ cell tumours were detected in the completely embedded specimen. Fluorescence in situ hybridization (FISH) examination revealed excess genetic material of chromosome 12p (isochromosome 12p) (Figure 6). This finding was considered as evidence for a somatic type malignancy occurring on the basis of a germ cell tumour metastasis in the retroperitoneum rather than a primary soft tissue tumour.

## 3. Discussion

Clinically, the case presented here is noteworthy because of several features that are uncommon but otherwise characteristic in patients with testicular GCT, and professionals caring for testis cancer patients should be aware of these. One feature is the excessive thrombosis of the vena cava extending into both iliac veins. As documented in the present patient, such events are pathogenetically associated with abdominal metastases compressing the vena cava, reducing blood flow velocity, and thus precipitating intravascular clotting [19–21]. Another peculiarity is the relapse of disease occurring as late as nine years after primary treatment. Late relapses of GCT are rare but may occur in about 2% of patients and chemotherapy is thought to be associated with these events [22, 23].

However, the most striking feature of the present case is the histology of the resected retroperitoneal mass. As outlined before, somatic type malignancies encompass a wide variety of malignant neoplastic histologies with sarcoma and carcinoma representing the most prevalent types [6]. Among the less frequently encountered histologies are PNET, haematologic neoplasms, and undifferentiated tumours [4]. Malignant peripheral nerve sheath tumours as seen in our patient are among the least common variants with only 4 cases reported so far [6, 13].

The pathogenesis of STM is poorly understood. The traditional theory forwarded by Mostofi and Sobin suggested mesenchymal elements within teratomas to give rise to malignant transformation [24]. In fact, STM is located in direct vicinity to teratoma in most of the cases either in primary testicular tumours or in metastatic deposits, and, accordingly, the old name teratoma with malignant transformation was coined. However, whether STM directly originates from teratoma remained unknown. Thus, a competing pathogenetic theory was forwarded postulating STM to originate from primitive totipotent germ cells [25]. Recently, the derivation of STM from the blastematous stroma of yolk sac tumours was suggested [26]. Finally, the rather frequent finding of STM in metastases surgically resected after chemotherapy [27, 28] suggested a selection process of chemotherapy-resistant cell populations to be another pathogenetic factor.

Our case is unique because neither teratoma nor yolk sac tumour had been documented at the time of first presentation. The primary tumour consisted of pure seminoma and reevaluation of the histologic sections did not reveal the presence of nonseminomatous elements. The occurrence of STM in a metastasis from pure testicular seminoma has never been reported before. Malagón et al. briefly mentioned a case of primary mediastinal seminoma containing components of a malignant nerve sheath tumour [29]; however, that particular constellation is probably different from primary testicular seminoma biologically. In our patient, evidence for the origin of the neurofibroma with transition to the MPNST from the previous GCT derived from the documentation of isochromosome 12p with FISH technique. Isochromosome 12p is characteristic of GCT [30] and it is retained in most of the STMs deriving from GCT, though lack of isochromosome 12p may not exclude the diagnosis of GCT [31]. In addition, the finding of mature bone within the tumour could be interpreted as residual teratoma component, thus arguing in favour of the GCT origin.

Three hypotheses would explain the findings in our patient. The first hypothesis assumes that the primary testicular seminoma as well as the metastasis at first presentation would have contained some foci of teratoma or even yolk sac tumour that escaped histological detection due to their small size. The minuscule foci of teratoma persisted at the site of the metastasis after chemotherapy despite radiological complete remission and gave rise to the full-blown STM at the time of clinical late relapse. The second hypothesis would suggest development of somatic type malignancy from seminoma cells. This pathway had not been conceived before and the transition from fully differentiated seminoma cells to completely dissimilar nongerm cell histologies would be rather enigmatic. The third hypothesis would suggest the presence of totipotent germ cells at the site of the primary metastasis that survived the first chemotherapy and gave rise to differentiation into teratoma and the STM. However, in the aggregate, no particular proof of any specific pathway can be given and the pathogenesis of the STM in our patient with seminoma remains indeterminate.

## 4. Conclusion

Development of somatic type malignancy from germ cell tumours is a rare event and its pathogenesis is still poorly understood. The present case supports the contention that conventional chemotherapy is ineffective in STM and that surgery is the mainstay of cure. The present case is unique because of the unprecedented origin of STM from pure seminoma generating one novel pathogenetic hypothesis of STM.

## Abbreviations

GCT: Germ cell tumour
STM: Somatic type malignancy
PNET: Primitive neuroectodermal tumours
FISH: Fluorescence in situ hybridization
CT: Computerized tomography
bHCG: Beta human chorionic gonadotropin
AFP: Alpha fetoprotein
LDH: Lactate dehydrogenase
MPNST: Malignant peripheral nerve sheath tumour.
